# Deciphering Immunosenescence From Child to Frailty: Transcriptional Changes, Inflammation Dynamics, and Adaptive Immune Alterations

**DOI:** 10.1111/acel.70082

**Published:** 2025-04-26

**Authors:** Wenpu Lai, Qiuyue Feng, Wen Lei, Chanchan Xiao, Juan Wang, Yi Zhu, Lipeng Mao, Yue Zhu, Jiacheng He, Yangqiu Li, Hao Wang, Zhenhua Li, Guobing Chen, Oscar Junhong Luo

**Affiliations:** ^1^ Department of Anesthesiology The First Affiliated Hospital, Jinan University Guangzhou China; ^2^ Key Laboratory for Regenerative Medicine of Ministry of Education, Institute of Hematology School of Medicine, Jinan University Guangzhou China; ^3^ Department of Systems Biomedical Sciences, School of Medicine Jinan University Guangzhou China; ^4^ Department of Microbiology and Immunology, School of Medicine, Institute of Geriatric Immunology, School of Medicine Jinan University Guangzhou China; ^5^ Musculoskeletal Pain Rehabilitation Department The Fifth Affiliated Hospital of Zhengzhou University Zhengzhou China; ^6^ Department of Physics & Astronomy University College London London UK; ^7^ Key Laboratory of Viral Pathogenesis & Infection Prevention and Control (Jinan University), Ministry of Education Guangzhou China

**Keywords:** frailty, immunosenescence, leukocytes, mononuclear, single‐cell gene expression analysis, transcriptome

## Abstract

Aging induces significant alterations in the immune system, with immunosenescence contributing to age‐related diseases. Peripheral blood mononuclear cells (PBMCs) offer a convenient and comprehensive snapshot of the body's immune status. In this study, we performed an integrated analysis of PBMCs using both bulk‐cell and single‐cell RNA‐seq data, spanning from children to frail elderlies, to investigate age‐related changes. We observed dynamic changes in the PBMC transcriptome during healthy aging, including dramatic shifts in inflammation, myeloid cells, and lymphocyte features during early life, followed by relative stability in later stages. Conversely, frail elderly individuals exhibited notable disruptions in peripheral immune cells, including an increased senescent phenotype in monocytes with elevated inflammatory cytokine expression, heightened effector activation in regulatory T cells, and functional impairment of cytotoxic lymphocytes. Overall, this study provides valuable insights into the complex dynamics of immunosenescence, elucidating the mechanisms driving abnormal inflammation and immunosuppression in frailty.

## Graphical abstract

Integrated analysis of bulk‐cell and single‐cell RNA‐seq data from childhood to frailty revealed nonlinear transcriptomic changes in peripheral blood mononuclear cells, marked by dramatic early‐life shifts in inflammation, myeloid cells, and lymphocyte features, followed by stability in healthy elderly individuals. In contrast, frail elderly individuals showed disrupted immune profiles, characterized by heightened monocyte‐driven inflammation, regulatory T‐cell activation, and impaired cytotoxic lymphocyte function.

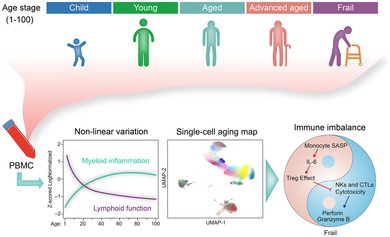

**Abbreviations**
CTL: cytotoxic T lymphocytes
DEGs: differentially expressed genes
DE‐SWAN: Differential Expression‐Sliding Window Analysis
GEO: Gene Expression Omnibus
GO: Gene Ontology
KEGG: Kyoto Encyclopedia of Genes and Genomes
PB: peripheral blood
PBMCs: peripheral blood mononuclear cells
PCC: Pearson correlation coefficient
RNA‐seq: RNA sequencing
SASP: senescence‐associated secretory phenotype
scRNA‐seq: single‐cell RNA sequencing
scTCR‐seq: single‐cell T‐cell receptor sequencing
Treg: regulatory T cell

## 1 Introduction

The immune system, essential for safeguarding against pathogens and preserving internal balance, undergoes age‐related alterations termed immunosenescence (Nikolich‐Žugich 2018). The immunosenescence process involves structural alterations in immune organs and dysfunctions in innate and adaptive immune functions, leading to reduced responsiveness to infections, vaccines, and an increased risk of chronic diseases and tumors (Desdín‐Micó et al. 2020; Fane and Weeraratna 2020). Understanding the mechanisms of immunosenescence is crucial for addressing the challenges of aging and improving the health status of elderly individuals.

Immunosenescence encompasses a range of changes, spanning alterations in immune cell counts and functionality, inflammation levels, immune response patterns, and the physiology of immune organs. A prominent feature of immunosenescence is inflammaging, a systemic chronic low‐grade inflammation state characterized by elevated inflammatory markers in the bloodstream, and is considered a key factor in aging (Liu et al. 2023). Senescent cells exhibit a unique phenotype known as the senescence‐associated secretory phenotype (SASP), characterized by the secretion of numerous soluble factors, including interleukin‐1 (IL‐1), IL‐6, IL‐8, IL‐13, IL‐18, tumor necrosis factor (TNF), and matrix metalloproteinase (Bruunsgaard et al. 2003; Ershler and Keller 2000). Consequently, increased levels of various inflammatory mediators are often detected in the serum of elderly individuals, which is closely associated with immune system dysregulation and the onset and progression of chronic diseases (Ferrucci and Fabbri 2018). Concentrations of circulating SASP‐related proteins were utilized to investigate and predict mortality in older adults (St Sauver et al. 2023).

Peripheral blood (PB) is frequently utilized in the research of immunosenescence; previous studies using PB samples have revealed age‐related changes in gene expression, proportion of cellular components, protein levels, and DNA methylation (Arthur et al. 2021; Harries et al. 2011; Jones et al. 2015; Lehallier et al. 2019; Luo et al. 2022; Terekhova et al. 2023; Zhu et al. 2023). Analyses of the relative abundance of immune cells in adults have enabled the construction of a high‐dimensional trajectory describing individual immune states, which accurately predicts overall mortality in adults (Alpert et al. 2019). However, most studies have focused on a limited age range (between 20 and 85 years old) (Arthur et al. 2021; Terekhova et al. 2023; Zhu et al. 2023), which were difficult to match the increasing life expectancy and aging population. Thus, investigation across a wider age range is necessary to achieve a more comprehensive understanding of immunosenescence.

Here, we integrated bulk‐cell and single‐cell RNA sequencing (RNA‐seq) data from a broad age range (1–99 and 1–100 years old, respectively) to probe the transcriptomic and cellular dynamics of peripheral blood mononuclear cells (PBMCs) from children to healthy and frail elderlies. We also validated these findings with flow cytometry and plasma protein quantification. The results revealed nonlinear dynamic changes in the PBMCs transcriptome with age, characterized by dramatic shifts in features, such as cytotoxicity, inflammation, myeloid cells, and lymphocytes occurring early in life, followed by a relatively slower and stable progression in healthy elderly individuals. In contrast, unhealthy aging (i.e., frailty) exhibited significant disruptions in peripheral immune cells, characterized by increased inflammation and immunosuppression.

## 2 Methods

### 2.1 Integration of Bulk‐Cell RNA‐Seq Datasets

This study compiled bulk‐cell RNA‐seq data from 93 healthy individuals from published literature (Table S1), including GSE206284, GSE166253, and GSE162562 from the Gene Expression Omnibus (GEO) database (https://www.ncbi.nlm.nih.gov/geo/). Additionally, bulk‐cell RNA‐seq data from PBMCs of 88 healthy individuals were newly generated. These datasets were combined to create a comprehensive bulk‐cell RNA‐seq dataset covering 181 healthy individuals.

### 2.2 Collection and Treatment of Blood Samples

In this study, all procedures performed involving human participants were under the ethical standards of the ethics committee and with the 1964 Declaration of Helsinki and its later amendments or comparable ethical standards. This study was approved by the Ethics Committee at the First Affiliated Hospital of Jinan University (Guangzhou, China) (JNUKY‐2023‐0047). Written consents were obtained. Participants were not compensated. Individuals who met the following criteria were excluded: progressive malignancy, acute progressive disease within 2 weeks, Alzheimer's disease, cognitive impairment, mental and neurological disorders, and other circumstances in which the researcher considered it inappropriate to participate in the study. Fresh EDTA‐anticoagulated PB from 88 donors were collected, which were processed within less than 24 h after collection. PBMCs are isolated from PB of healthy donors using Ficoll (StemCell Technologies, Vancouver, Canada) following the manufacturer's protocol. Total RNA from PBMCs was isolated by MolPure TRIeasy Plus Total RNA Kit (YEASEN, Shanghai, China). Then the quality of RNA was checked by the Nanodrop (Thermo Fisher Scientific, Waltham, USA) or the Agilent Bioanalyzer 2100 system (Agilent Technologies, Santa Clara, USA).

### 2.3 Library Preparation for Bulk RNA Sequencing

A total amount of 1 μg total RNA per sample was used as input material for the library preparation of bulk RNA sequencing. Sequencing libraries were generated using Hieff NGS Ultima Dual‐mode mRNA Library Prep Kit for Illumina (YEASEN, Shanghai, China) following the manufacturer's protocol. Briefly, mRNA was purified from total RNA by mRNA capture beads. Fragmentation was carried out using Frag/Prime Buffer under 94°C. First strand cDNA was synthesized using Strand Specificity Reagent and 1st Strand Enzyme Mix. Second strand cDNA synthesis combined with end prep and dA‐tailing was subsequently performed by 2nd Strand Enzyme Master Mix in 2nd Strand Buffer (dNTP). After complete adapter ligation, cDNA fragments with inserted DNA size from 350 to 450 were purified with Hieff NGS DNA Selection Beads (YEASEN, Shanghai, China). Then 2× Super Canace II High‐Fidelity Mix was used with size‐selected, adaptor‐ligated cDNA for library amplification. At last, PCR products were purified with Hieff NGS DNA Selection Beads (YEASEN, Shanghai, China) to obtain the library for RNA‐seq.

### 2.4 Library Quality Control and Sequencing

After library preparation, quantification of the library was detected by Qubit2.0 Fluorometer (Thermo Fisher Scientific, Waltham, USA). Then the library was diluted to 1.5 ng/μL and its size was assessed on the Agilent Bioanalyzer 2100 system (Agilent Technologies, Santa Clara, USA). Passed libraries were pooled according to the requirements of effective concentration and target data volume, and then sequenced on Illumina NovaSeq 6000 for 150‐bp paired‐end reads.

### 2.5 Processing of Raw RNA‐Seq Data

Raw sequencing reads were assessed using FastQC, followed by trimming with Trimmomatic (http://www.usadellab.org/cms/index.php?page=trimmomatic). The trimmed reads were aligned to the reference genome GRCh38 using HISAT2 (https://github.com/DaehwanKimLab/hisat2), and the resulting SAM files were converted to sorted BAM files with SAMtools (http://samtools.sourceforge.net). Gene expression was quantified using FeatureCounts with exon‐level summarization (http://subread.sourceforge.net).

### 2.6 Downstream Analyses of RNA‐Seq Data

Batch effect removal was performed using Combat (Johnson et al. 2007), followed by downstream analyses. The effectiveness of batch effect removal was evaluated by comparing the distribution of samples, median expression levels, and Pearson correlation coefficient (PCC) before and after batch correction. Only genes expressed in at least 75% of samples were kept for analysis. We first aimed to identify genes with linear expression changes across age (Lehallier et al. 2019). The following model was implemented:

$$\text{Gene expression}\sim \alpha +\beta_{1}\mathrm{age}+\beta_{2}\mathrm{sex}+\varepsilon .$$

Log‐normalized transcripts per million (TPM) were used for expression values as recommended for differential expression analyses. α represents the y‐intercept, *β* values represent the associated slope with the variable of interest, and εrepresents residual error. Sex was included as a covariate to account for variations in sex composition of the cohort across age. Linear models were generated using the R package stats function *lm()*. Type II sum of squares were calculated using the *Anova()* function of R package car. *p* values were adjusted for multiple comparisons using the Benjamini–Hochberg procedure. Thresholds of 0.05 for adjusted *p* value were used to determine differentially expressed genes (DEGs). Thresholds of 0.005 for *β* of DEGs were used to further enrichment analysis. R package Clusterprofiler (https://github.com/YuLab‐SMU/clusterProfiler) were used for Gene Ontology (GO) and Kyoto Encyclopedia of Genes and Genomes (KEGG) pathway enrichment analyses.

LOESS was employed to identify nonlinear patterns of gene expression with age. A LOESS regression of span 0.75 was fit to each gene using the *loess()* function of the R package stats. The predicted expression trajectories over age were then subdivided into 6 to 12 groups by hierarchical clustering via *hclust()* function from the R package stats. LOESS curves of average expression per age point in each cluster are also reported.

Differential Expression‐Sliding Window Analysis (DE‐SWAN) was implemented to identify more transient gene expression changes with age (Lehallier et al. 2019). The following model was used:

$$\text{Gene expression}\sim \alpha +\beta_{1}I_{k\;\mathrm{low}/\text{high}}\mathrm{age}+\beta_{2}\mathrm{sex}+\varepsilon$$

$I_{k\;\mathrm{low}/\text{high}}$ represents the binarization of age binned above and below *k* centers. Centers with windows of ±10 years from age of 10 to 90 were used. Top Significant DEGs were identified with thresholds of 0.01 for adjusted *p* value and 0.5 for β.

### 2.7 Collection of Public PBMCs scRNA‐Seq Dataset

We assembled scRNA‐seq data from 47 healthy individuals with known age information, along with data from five frail elderly individuals, sourced from published literature (Table S6). The datasets included GSE157007, GSE158055, GSE168732, GSE181279, GSE206284, GSE206295 from the GEO database, and the SC2018 dataset (http://gerg.gsc.riken.jp/SC2018/). Among the scRNA‐seq datasets, 39 samples had matching single‐cell T‐cell receptor sequencing (scTCR‐seq) data. scRNA‐seq data were categorized into five groups on the basis of age and status: child (1–11 years), young (23–58 years), aged (60–72 years), advanced‐aged (77–100 years), and frail elderly. Elderlies classified into the frail group were based on the frailty assessment according to the original publication (Luo et al. 2022).

### 2.8 Integration and Annotation of scRNA‐Seq Data

Following quality control based on mapped features count and mitochondrial reads for each dataset, we utilized SCALEX (Xiong et al. 2022), a neural network designed for rapid integration of multi‐batch scRNA‐seq data, to integrate and project the data. To evaluate the batch correction performance for sRNA‐seq data, we used conventional processing methods (data normalization, standardization, and principal component analysis for dimensionality reduction) as standard data quality control and assessed the effectiveness using the Integration Local Inverse Simpson's Index (Korsunsky et al. 2019).

Subclusters within major cell types, T, Natural Killer (NK), B, and myeloid cells, were identified through a second round of clustering. The definition of T‐cell subtypes included naïve, memory, and effector T cells according to specific genes, such as *SELL*, *TCF7*, *LEF1*, *GZMK*, *GZMB*, and *PRF1*. Regulatory T cells (Treg) were defined by *FOXP3* and *IL2RA*, whereas mucosal‐associated invariant T cells (MAIT) and Gamma‐delta T cells (γδ T) were characterized by *TRAV1‐2*, *SLC4A10*, *TRDV2*, and *TRGV9*. NK cells were initially clustered by *NCAM1* (CD56) and *FCGR3A* (CD16), then were further annotated into terminal differentiated (*GZMB*, *PRF1*), memory‐like (*KLRC2*, *HLA‐DRB1*), naïve (*SELL*, *IL7R*), and type I interferon‐responsive (*CD69*, *IFNG*) NK cells (Guo et al. 2022; Smith et al. 2020). B‐cell subtypes were defined by the expression of *IGHD*, *CD27*, and *XBP1*, for naïve, memory B cells, and plasma cells, respectively. Among B cells, the naïve B‐cell subtypes, characterized by elevated expression of *TCL1A* and *SOX4*, are considered a population of transition B cells (Morgan and Tergaonkar 2022). Memory B cells are further categorized into three groups: IgM memory, classical memory, and age‐related B cells, which highly expressed *ITGAX* (Morgan and Tergaonkar 2022). Myeloid cell subtypes included CD14^+^ monocytes, intermediate monocytes, CD16^+^ monocytes, classical dendritic cells (cDC), and plasmacytoid dendritic cells (pDC), marked by *CD14*, *CD16*, *CD1C*, and *CLEC4C* (Villani et al. 2017). The analysis involved identifying inflammatory and antigen‐presenting functions in CD14^+^ monocytes with high expression of *S100A8* and *HLA‐DRA* (Villani et al. 2017). Cells highly expressing *MALAT1* were defined as cells undergoing cell death (Zheng et al. 2021), and those expressing signature genes of two or more immune cell types were designated as doublets. Red blood cells (RBC) and megakaryocytes were identified by *HBB*, *HBA2*, *PF4*, and *PPBP*.

DEGs analysis for scRNA‐seq was performed using the Python package Scanpy (https://github.com/scverse/scanpy), with parameters set as *p.adj*. < 0.05 and log_2_[fold‐change] > 0.25. Using the *tl.score_genes*() function in Scanpy, we calculated the functional gene set scores for each single cell. The SASP functional score of monocytes was calculated on the basis of the following genes: *CCL2*, *CCL3*, *CCL4*, *CCL5*, *CXCL1*, *CXCL2*, *CXCL3*, *CXCL8*, *CXCL10*, *HMGB1*, *ICAM1*, *IL18*, *IL1B*, *IL6*, *TNF*, *MMP9*, *MIF*, *NRG1*, *EREG*, and *AREG*.

### 2.9 Analysis of scTCR‐Seq Data

Only TCR sequences associated with cells annotated as T cells by scRNA‐seq were retained. The R package STARTRAC (https://github.com/Japrin/STARTRAC) was employed to quantify TCR clonal expansion, Gini coefficients, and cell state transition potential for each T‐cell subtype.

### 2.10 Inference and Analysis of Cell–Cell Communication

We evaluated the intercellular communication between different cell types of healthy elderly and frail elderly individuals using the R package CellChat (Jin et al. 2021). CellChat takes gene expression data as input and models the probability of intercellular communication by integrating gene expression with known databases of interacting ligands, receptors, and their cofactors. The data from healthy advanced‐aged and frail elderly were subsampled to 5000 cells each, excluding doublets, RBC, megakaryocytes, and subtype with fewer than 10 cells or high expression of *MALAT1*. Subsequently, we conducted an analysis of intercellular communication between the two groups using the default workflow.

### 2.11 NicheNet Analysis

NicheNet is a computational method that uses prior knowledge on ligand–receptor, signal transduction, and gene regulatory networks to prioritize ligand–receptor pairs that could regulate the expression of a gene set of interest (Browaeys et al. 2020). The gene set was determined as the upregulated genes (with three different log_2_[fold‐change] thresholds: 0.5, 0.75, and 1) in frailty versus advanced‐aged for CD4_Treg cells. Additionally, to further investigate the potential ligand‐specific regulation of CD4_Treg cells from frail donors, we also performed NicheNet analysis on advanced‐aged individuals and all CD4_Treg cells. The gene set for CD4_Treg cells in advanced‐aged individuals was determined on the basis of upregulated genes in frailty vs. advanced‐aged with a log_2_[fold‐change] threshold of 0.75, while the gene set for all CD4_Treg cells consisted of genes expressed in at least 50% of the cells. The analysis was limited to receptors that were expressed in at least 10% of the cells of CD4_Treg cells in corresponding sample groups.

### 2.12 ELISA Assays

In total, Human IFN‐γ, IL‐6, IL‐10, Granzyme B, CRP, TNF‐α, IL‐1β, TGF‐β1, IL‐10, and Anxa1 were detected using ELISA in 14 plasma samples, including four frail and 10 healthy old donors, in accordance with the manufacturer's instructions (Tables S8, S9). The frailty index of these 14 old donors was assessed as previously described (Luo et al. 2022), which is based on the frailty index assessment model created by the Rockwood laboratory (Searle et al. 2008). After the Frailty index test, donors over 65 years old with a score greater than 0.2 were assigned to the “frail” group.

### 2.13 Flow Cytometry

PBMCs were retrieved from −80°C and revitalized in a 37°C water bath. The cells were washed once with sterile PBS, filtered through a 70‐μm filter, and formulated into a 2 × 10^6^ cells/mL suspension. Subsequently, the cells were re‐suspended in FACS buffers (2% FBS, 1 × PBS, 2 mM EDTA, and 0.04% sodium azide) at a density of 2 × 10^6^ cells/mL. After being washed twice, the cells were surface‐stained with fluorescent dye‐coupled monoclonal antibodies CD3‐Pacific Blue (clone SP34‐2, BD Biosciences, Franklin Lakes, USA), CD4‐BV510 (clone SK3, BD Biosciences, Franklin Lakes, USA), CD8‐RB545 (clone RPA‐T8, BD Biosciences, Franklin Lakes, USA), CD25‐BV480 (clone M‐A251, BD Biosciences, Franklin Lakes, USA), CD45RA‐BV786 (clone HI100, BD Biosciences, Franklin Lakes, USA), CD45RO‐APC‐H7 (clone UCHL1, BD Biosciences, Franklin Lakes, USA), CD278‐PE (clone DX29, BD Biosciences, Franklin Lakes, USA), PD‐1‐PE‐CY7 (clone EH12.1, BD Biosciences, Franklin Lakes, USA), KLRG1‐BV750 (clone Z7‐205.rMAb, BD Biosciences, Franklin Lakes, USA), CD57‐BB515 (clone NK‐1, BD Biosciences, Franklin Lakes, USA), and CD62L (clone DREG‐56, BD Biosciences, Franklin Lakes, USA) at 4°C for 15 min (Table S10).

Afterward, the above cells were washed twice with FACS buffer and fixed with 2% paraformaldehyde for 40 min. For intracellular staining, a cytofix/cytoperm buffering kit purchased from BD Pharmingen was utilized, and the cells were processed in accordance with the manufacturer's instructions. The cells were intracellularly stained with fluorescent dye‐coupled monoclonal antibody GZMB‐RB705 (clone GB11, BD Biosciences, Franklin Lakes, USA). The above cells were washed twice with FACS buffer, stained with Live/Dead‐FVS620 (BD Biosciences, Franklin Lakes, USA) prior to up flow cytometry. Finally, the samples were analyzed on Cytek flow cytometry (Cytek Aurora) and processed using FlowJo‐V10.8 software (BD Biosciences, Franklin Lakes, USA).

### 2.14 Statistics Analysis

All statistical analyses were performed using R (version 4.1.0) and Python (version 3.7.3), including the Kruskal–Wallis test, Wilcoxon rank sum test, ANOVA test, and *t*‐test. *p* < 0.05 was considered statistically significant, unless stated otherwise.

## 3 Results

### 3.1 Dynamic Alterations in PBMCs Transcriptome Through Healthy Aging

**FIGURE 1:**
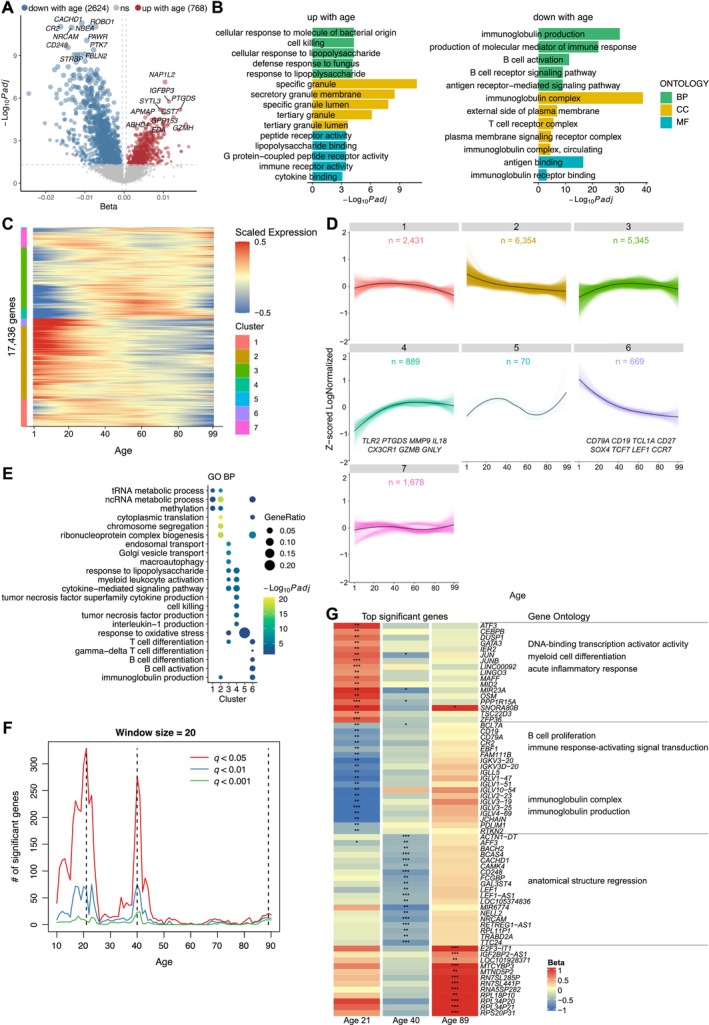
Age‐associated changes in PBMC transcriptome. (A) Volcano plot showing changes in gene expression of PBMCs with age. Linear models, adjusted for age and sex, were tested using the F‐test. (B) Significantly enriched GO terms for the upregulated (left) and downregulated (right) genes with age, respectively. The top five terms per ontology category are presented. BP: biological process; CC: cellular component; MF: molecular function. (C) Heatmap of gene expression trajectory with age, estimated by LOESS. Unsupervised hierarchical clustering grouped genes with similar trajectories into seven clusters. (D) Line graphs showing the gene expression trajectories along age, separated as clusters defined by unsupervised hierarchical clustering. Black line in each panel represents the average trajectory for each cluster; the number of genes is shown for each cluster. Representative genes are shown for Clusters 4 and 6. (E) GO biological process enrichment for genes in each cluster of Figure 1D. (F) Line graphs showing the number of genes differentially expressed with age. Three local peaks at the ages of 21, 40, and 89 years were identified by DE‐SWAN, with different *q*‐value cutoffs represented in different colors. (G) Left: Top significant DEGs identified by DE‐SWAN at age of 21, 40, and 89 years. Blue and red represent local decrease and increase, respectively. *q < 0.05, **q < 0.01, ***q < 0.001. Right: Significantly enriched GO terms for the respective genes. *, ** and *** are used to indicate the statistical significance (q‐value) in Figure 1G.

We integrated PBMCs bulk‐cell RNA‐seq data from 181 healthy individuals (88 in‐house generated and 93 public datasets, respectively), creating a comprehensive cohort spanning age from 1 to 99 years (Figure S1A and Table S1). The integrated data, evenly distributed across age and gender (Figure S1B,C), underwent normalization and batch effect mitigation (Figure S1D). After batch effect correction, there was no obvious batch effect observed among the samples (Figure S1D–F). Employing linear models, we explored age‐related gene expression changes (Methods). It identified 769 genes upregulated with age, including *PTGDS* and *GZMH*, and 2624 genes downregulated with age, including *CR2* and *CD248* (Figure 1A and Table S2). GO and KEGG pathway enrichment analysis indicated a decline in lymphocyte characteristics and downregulation of pathways with age, such as “B cell receptor signaling pathway,” “immunoglobulin production,” and “T cell receptor complex” (Figure 1B right and Figure S1G). Conversely, pathways related to cytokines and cytotoxicity were found to be upregulated with age, such as “Cytokine receptor interaction” and “cell killing” (Figure 1B left and Figure S1G).

To understand transcriptomic trends through human aging, we applied LOESS smoothing to gene expression versus age (Figure S1H). Hierarchical clustering identified distinct age‐related gene expression patterns: in particular, Clusters 4 (*TLR2*, *PTGDS*, *MMP9*, *IL18*, *CX3CR1*, *GZMB*, *GNLY*, etc.) and Cluster 6 (*CD79A*, *CD19*, *TCL1A*, *CD27*, *SOX4*, *TCF7*, *LEF1*, *CCR7*, etc.) exhibited clear age‐related improvement or decline, and were enriched in cytotoxicity, inflammation, myeloid cell features, and lymphocyte characteristics, respectively (Figure 1C–E and Figure S1I; Table S3). Notably, these expression patterns underwent rapid changes in early life, gradually slowed down, and stabilized in healthy old donors.

Additionally, using DE‐SWAN, we identified gene expression differential peaks at 21, 40, and 89 years of age, each associated with unique transcriptomic patterns (Figure 1F,G; Tables S4, S5). At 21 years of age, upregulated genes were enriched for inflammation and myeloid cell functions, whereas downregulated genes were linked to B‐ and plasma cell functions. At 40 years, downregulated genes, including *LEF1* and *CD248*, were enriched for “anatomical structure regression,” aligning with previously reported negative aging effects (Harries et al. 2011). Interestingly, the specifically up‐ or downregulated genes at 21 and 40 years stabilized by the age of 89, and only upregulation of ribosomal genes were found in advanced‐aged individuals (Zhu et al. 2023).

### 3.2 Dynamic Alterations in PBMC Composition and TCR Repertoire With Age

**FIGURE 2:**
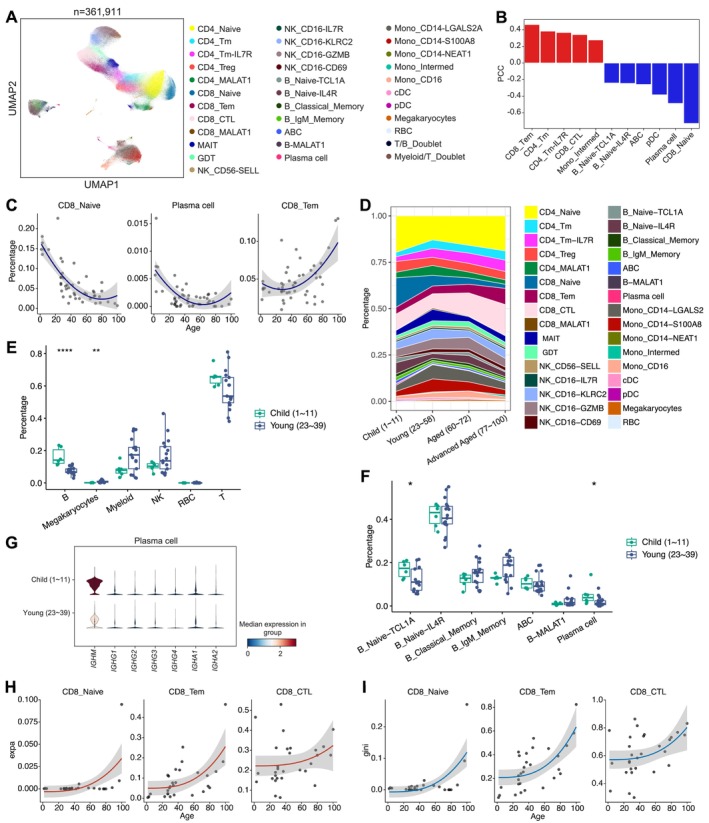
Age‐associated changes in PBMC by scRNA‐seq and scTCR‐seq. (A) UMAP (Uniform manifold approximation and projection) visualization of PBMC single cell clusters. Different cell types were depicted with distinct colors. (B) PCC of proportion of cell type with age according to PBMCs scRNA‐seq data from healthy individuals. Only cell types with the absolute values of PCC > 0.2 are shown. (C) Scatter‐plots of the proportion of cell type (*y*‐axis) in PBMCs (according to scRNA‐seq analysis) vs. age (*x*‐axis) of the individual. Only cell types with absolute values of PCC > 0.4 between cell type proportion and age are shown. Fitted lines by polynomial function and confidence intervals (gray shaded area) are shown. (D) Variation of proportion of each defined cell type across age groups. (E) Boxplots for comparing the proportion of major cell type between child and young adult sample groups. **p < 0.01, ****p < 0.0001. Wilcoxon rank sum test (two‐sided). (F) Boxplots for comparing the proportion of subtypes of B cells between child and young adult sample groups. *p < 0.05. Wilcoxon rank sum test (two‐sided). (G) Violin plots showing the immunoglobulin‐related gene expression levels of plasma cells between child and young adult sample groups. (H) Scatter plots of TCR clonal expansion (y‐axis) vs. age (x‐axis) for selected T‐cell subtype. Fitted lines by polynomial function and confidence intervals (gray shaded area) are shown. (I) Same as H, but for Gini coefficient of TCR repertoire vs. age.

We integrated scRNA‐seq data from 47 healthy individuals covering 1 to 100 years old, creating a PBMCs atlas of over 360,000 single cells (Figure 2A and Figure S2A,B; Table S6). Six major cell types were defined on the basis of their respective marker genes (Figure S2C,D). After processing by SCALEX, the scRNA‐seq data showed no obvious batch effects (Figure S2A–C,E). In addition, 32 distinct cell subtypes (excluding doublet clusters) were further defined on the basis of their respective marker genes (Figure 2A and Figure S2F–I). Cell types associated with aging include memory T‐cell subsets (CD8_Tem, CD4_Tm, CD4_Tm‐IL7R) and the cytotoxic T lymphocytes (CTL) subset CD8_CTL, which exhibited a positive correlation with age. Conversely, the abundance of CD8_Naive, plasma cell, pDC, and B‐cell subsets showed a negative correlation with age (Figure 2B–D). Notably, the population of plasma cells and CD8_Naive cells, most strongly anti‐correlated with age, especially through 1 to 60 years old, maintained stability in late healthy aging, consistent with the dynamics observed in the bulk‐cell transcriptome (Figure 2C).

Considering the significant B‐cell‐related transcriptional changes observed in bulk RNA‐seq during the transition from childhood to adulthood, we conducted a comparative analysis between children (1–11 years old) and young adults (23–39 years old). We found a significant decrease in the overall proportion of B cells in young adults compared with children (Figure 2E). Subtypes, such as B_Naive‐TCL1A cells, characterized by transitional B‐cell features, and plasma cells, exhibited a marked decline in their proportions within the B‐cell population in young adults, accompanied by reduced *IGHM* expression in plasma cells (Figure 2F,G). Additionally, although all B‐cell subpopulations in young adults, except for B_IgM_Memory and B‐MALAT1 cells, showed a significant decrease in proportions within PBMCs, the MAIT cells, innate‐like T cells, and the myeloid cell subset Mono_CD14_LGALS2 cells demonstrated an increased proportion compared with children (Figure S2J). Thus, B‐cell‐related transcriptional changes in bulk RNA‐seq during the transition from childhood to adulthood may be contributed to by both the variation in proportion of B‐cell subpopulations and functional changes (Ciocca et al. 2021).

In scTCR‐seq analysis, CD8_Tem (CD8^+^ effector memory T) and CD8_CTL cells displayed obvious clonal expansion, and they had the highest functional transition potential (Figure S3A,B). Furthermore, most T‐cell subtypes, except CD4_Treg (CD4^+^ regulatory T) and CD8_MALAT1 (*MALAT1* ^high^ CD8^+^ T) cells, exhibited decreased TCR diversity (i.e., increased clonal expansion and Gini coefficients) beyond 60 years old (Figure 2H,I and Figure S3C,D), echoing a reported aging inflection point around the age of 65 (Naylor et al. 2005).

### 3.3 Heightened Inflammatory Response for PB of Frailty

**FIGURE 3:**
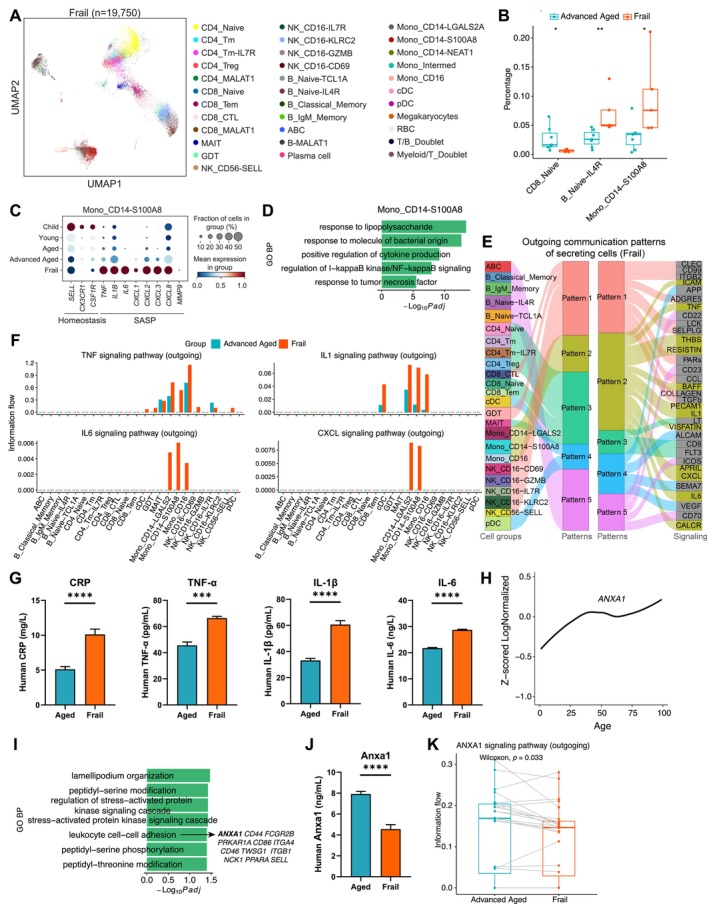
Heightened inflammatory response for PB of frail elderly elders. (A) UMAP visualization of scRNA‐seq data from PBMCs of frail individuals. Different cell types are depicted with distinct colors. (B) Boxplots for comparing the proportion of selected cell types between frail and healthy advanced‐aged individuals. *p < 0.05, **p < 0.01. Wilcoxon rank sum test (two‐sided). (C) Dotplots showing the expression of selected genes in Mono_CD14‐S100A8 cells from distinct age groups. The color and size of each dot represent the expression level and cell fraction expressing the genes, respectively. The same applies to the rest of this figure. (D) Significantly enriched GO biological process terms of upregulated genes in Mono_CD14‐S100A8 cells of frail individuals in comparison with healthy advanced‐aged individuals. (E) The inferred outgoing communication patterns of secreting cells for frailty, which show the correspondence between the inferred latent patterns and cell groups, as well as signaling pathways. The thickness of the flow indicates the contribution of the cell group or signaling pathway to each latent pattern. (F) Bar plots showing outgoing information flow of TNF, IL1, IL6, and CXCL signaling pathways for each cell type of advanced‐aged and frail donors. (G) Plasma protein levels of CRP, TNF‐α, IL‐1β, and IL6 for aged and frail donors. ***p < 0.001, ****p < 0.0001. T‐test (two‐sided). (H) Line graphs showing the *ANXA1* expression trajectories along age. (I) Significantly enriched GO biological process terms for genes positively correlated with *ANXA1* (PCC > 0.7) in bulk RNA‐seq samples from donors aged 75 and older. Genes enriched in leukocyte cell–cell adhesion are shown. (J) Same as G, but for Anxa1 protein. (K) Boxplots comparing the information flow of ANXA1 signaling pathways for each cell type from advanced‐aged and frail elderly individuals. Each paired dot corresponds to the two populations of the same cell type from the two sample groups. Paired Wilcoxon rank sum test for the same cell types of two groups (two‐sided).

To further explore the alterations in peripheral immune cells of unhealthy aging, we projected the PBMCs atlas onto frail (a pathological aging state) elderly individuals, comparing it with data from healthy advanced‐aged individuals (Figure 3A and Figure S4A). Frailty led to a significant decrease in CD8_Naive cells and a notable increase in Mono_CD14‐S100A8 (CD14^+^ *S100A8* ^high^ monocyte) and B_Naive‐IL4R cells (Figure 3B). Consequently, we proceeded to investigate the functional changes in these cell subsets with altered abundance between healthy and frail old individuals (Table S7). Compared with the healthy elderlies, frail donors exhibited enrichment of stress‐induced MAPK cascade and NF‐κB signaling pathways in B_Naive‐IL4R cells (Figure S4B,C). Additionally, CD8_Naive cells in frail individuals showed upregulation of T‐cell aging and effector T‐cell‐related genes, such as *KLRG1* and *GZMK*, with downregulation of *CCR7*, *IL7R*, *GPR183*, and *CXCR4*. These cells also exhibited increased activity in pathways related to apoptosis, DNA damage, NF‐κB signaling, and response to TNF when compared with healthy elderlies (Figure S4D,E). A similar decline in the proportion of naïve CD8^+^ T cells (CD45RA^+^CD62L^+^CD8^+^) was observed via flow cytometry in frail individuals from an additional sample cohort (Figure S4F–H).

In addition, Mono_CD14‐S100A8 cells demonstrated a decline in the expression of homeostatic genes and a gradual increase in inflammatory factor expression during healthy aging (e.g., *TNF, IL1B*, and *CXCL8*) (Figure 3C). However, in frail elderly individuals, these cells exhibited rapid upregulation of inflammatory factors, and monocytes ubiquitously exhibited notably higher SASP functional scores (Figure S4I), suggesting prominent SASP characteristics. Mono_CD14‐S100A8 cells were also enriched for pathway activities related to lipopolysaccharide and TNF response, and NF‐κB signaling in frail individuals (Figure 3D). Furthermore, cell–cell communication analysis revealed that inflammation‐related signaling pathways (TNF, IL1, IL6, and CXCL) in PBMCs were primarily driven by the monocyte subpopulations (Figure 3E), with an increase in their output in frail elderly individuals compared with healthy elderly individuals (Figure 3F). To assess the levels of inflammatory activation and cytokine concentrations in PB, we analyzed the expression of CRP, TNF‐α, IL‐1β, and IL‐6 proteins in an additional cohort by ELISA. Similarly, these protein levels were significantly elevated in frail individuals compared with healthy elderlies (Figure 3G). Interestingly, the anti‐inflammatory gene *ANXA1* exhibited increased mRNA expression with aging (Figure 3H), while genes with a PCC greater than 0.7 with *ANXA1* in bulk RNA‐seq data from healthy individuals aged 75 and older were enriched in GO terms like leukocyte cell–cell adhesion (Figure 3I). However, plasma protein levels of Anxa1 were significantly reduced in frail individuals compared with healthy elderlies (Figure 3J). This decrease was also evident in its signaling output in cell communication analysis (Figure 3K). These findings potentially suggested that *ANXA1* plays a role in anti‐inflammatory processes during healthy aging and frailty (Singh et al. 2024; Tampé et al. 2024).

### 3.4 Clonal Expansion and Effector Treg Cells in Frailty

**FIGURE 4:**
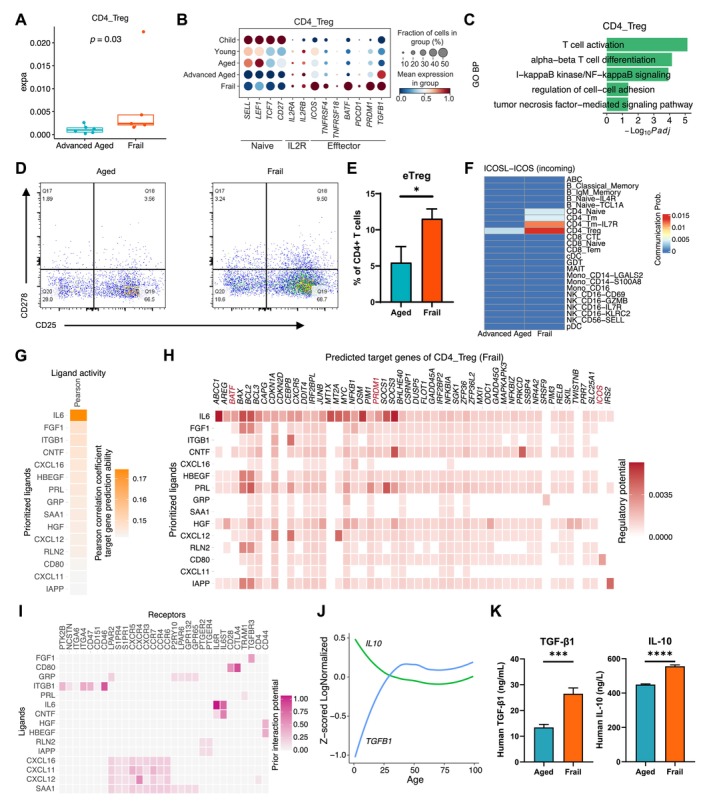
Activation of Treg cells in frailty. (A) Boxplots for comparing of TCR clonal expansion of CD4_Treg cells between frail and healthy advanced‐aged (age ≥ 75) individuals. Wilcoxon rank sum test (two‐sided). (B) Dotplots showing expression of selected genes in CD4_Treg cells from distinct age groups. (C) Significantly enriched GO biological process terms of upregulated genes in CD4_Treg cells of frail individuals in comparison with healthy advanced‐aged individuals. (D) The gating strategies of eTreg cells (CD278^+^CD25^+^CD4^+^ T cells) for aged and frailty. (E) Bar plot showing percentage for eTreg of CD4^+^ T cells in aged and frail donors. *p < 0.05. T‐test (two‐sided). (F) Incoming communication probability of ICOSL‐ICOS signal for each cell types of advanced‐aged and frail elderlies. (G) NicheNet analysis of the upregulated gene set in Treg cells (log_2_FC > 0.75) of frail compared with advanced‐aged donors. Heatmap shows the top 15 ligands with the highest predictive power for the gene expression in Treg cells of frail donors. The Pearson's correlation coefficient reflects the ability of each ligand to accurately predict the target genes in Treg cells. (H) Heatmap of NicheNet ligand–target matrix denoting the regulatory potential between top 15 ligands of DEGs (log_2_FC > 0.75) and target genes in Treg cells from frail donors. Gene related to eTreg cells are shown in red. (I) Potential receptors of Treg cells interacting with the top 15 ligands of Figure 4G. (J) Line graphs showing the *TGFB1* and *IL10* expression trajectories along age. (K) Plasma protein levels of TGF‐β1 (left) and IL‐10 (right) for aged and frail donors. ***p < 0.001, ****p < 0.0001. T‐test (two‐sided).

We observed an increase in CD4_Treg cell TCR clonal expansion and elevated expression of effector Treg (eTreg)‐related genes, such as *ICOS*, *BATF*, and *PRDM1*, within CD4_Treg cells of frail individuals, with these CD4_Treg cells showing expression enrichment in T‐cell activation and TNF‐mediated signaling pathways compared with healthy elders (Figure 4A–C). However, with healthy aging, apart from elevated expression of *TGFB1*, there was no obvious elevation in effector‐related genes of CD4_Treg cells, consistent with the absence of noticeable changes in CD4_Treg cells TCR diversity with age (Figure 4B and Figure S3C). Flow cytometry analysis revealed that the proportion of eTreg (CD25^+^CD278^+^CD4^+^ T) cells was also elevated in frail individuals (Figure 4D,E). Further investigation in frailty showed that the ICOSL‐ICOS signaling pathway, which supports eTreg maintenance (Raynor et al. 2015), was not only enriched in Treg cells but also in conventional CD4^+^ T‐cell subsets (Figure 4F and Figure S5A). Similarly, in frail donors, CD278^+^CD4^+^ T‐cell population increased, and conventional CD4^+^ T cells showed high expression of eTreg‐related genes (Figure S5B,C). Using NicheNet analysis, we found that IL‐6 was consistently identified as the top potential regulator of Treg characteristic genes across distinct thresholds in frail individuals, while it was not observed in the advanced‐aged group or in all Treg cells (Figure 4G and Figure S5D–F). IL‐6, through its receptor, potentially regulated a broad range of upregulated genes in Treg cells from frail individuals, including eTreg‐related genes, such as *BATF*, *PRDM1*, and *ICOS* (Figure 4H,I) (Raynor et al. 2015). In addition, we observed that the mRNA levels of two Treg‐associated immunosuppressive molecules, IL‐10 and TGF‐β1, remained relatively stable in healthy elderly individuals (age ≥ 50) (Figure 4J). However, in frail individuals, there was a significant increase in the plasma protein levels of these molecules (Figure 4K).

### 3.5 Suppression of Cytotoxic Function in Frailty

**FIGURE 5:**
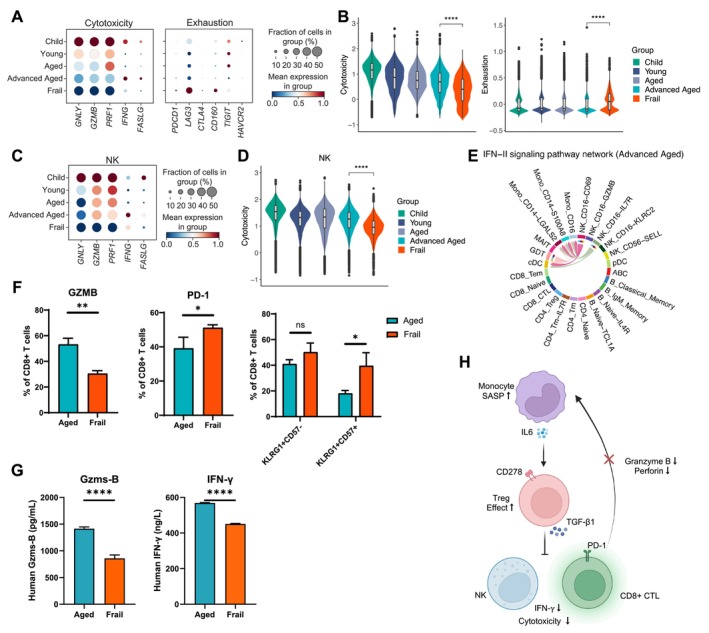
Suppression of cytotoxic function in frailty. (A) Dotplots showing expression of cytotoxicity and exhaustion‐related genes in CD8_CTL cells from distinct age groups. (B) Violin plots showing cytotoxicity (left) and exhaustion (right) functional scores of CD8_CTL cells across different age groups. ****p < 0.0001. Wilcoxon rank sum test (two‐sided). (C) Dotplots showing expression of cytotoxicity‐related genes in NK cells from distinct age groups. (D) Violin plots showing cytotoxicity functional scores of NK cells across different age groups. ****p < 0.0001. Wilcoxon rank sum test (two‐sided). (E) Chord diagram showing the IFN‐II signaling pathway activity among different cell types of PBMCs in advanced‐aged individuals. The signaling activities of the IFN‐II pathway among the analyzed cell types in the frail group were not identified; therefore, no chord diagram was produced. Also see Figure S5. (F) Bar plots showing the percentage for GZMB^+^, PD‐1^+^, KLRG1^+^CD57^−^, and KLRG1^+^CD57^+^CD8^+^ T cells in aged and frail donors from left to right. NS not significant, *p < 0.05, **p < 0.01. T‐test (two‐sided). (G) Plasma protein levels of Gzms‐B and IFN‐γ for aged and frail donors. ****p < 0.0001. T‐test (two‐sided). (H) Schematic representation of heightened inflammatory response and immunosuppression in peripheral immune cells of frail elderly individuals.

The shift toward an immunosuppressive phenotype in CD4^+^ T cells (Löhning et al. 2003), particularly Treg cells, in frail elderly individuals raises concerns about the potential alterations in cytotoxic cell function. We found that the expression of cytotoxicity‐related genes and overall functional scores in CD8_CTL and NK cells were decreased in frail individuals, while in the healthy elderly group, these declines were more gradual (Figure 5A–D). In contrast, the expression of exhaustion‐related genes and overall exhaustion scores in CD8_CTL cells increased (Figure 5A,B). Additionally, IFN‐II cell–cell interaction networks were enriched exclusively in healthy elderly individuals and were driven by outputs from NK cell subsets and γδ T cells (Figure 5E and Figure S5G). Flow cytometry also revealed a higher proportion of PD‐1^+^CD8^+^ T cells, and KLRG1^+^CD57^+^CD8^+^ T cells in frail individuals compared with old healthy donors, along with a lower proportion of GZMB^+^CD8^+^ T cells in frail individuals (Figure 5F). Similarly, Granzyme B and IFN‐γ had higher plasma protein levels in healthy elderly individuals compared with frailty (Figure 5G). These findings suggest that the frail individuals had a dysregulated peripheral immune state, where an imbalance between pro‐ and anti‐inflammatory responses was potentially driven by increased SASP‐monocytes and IL‐6‐induced Treg cells activation, leading to suppressed cytotoxic cell function and a reduced capacity to eliminate abnormal immune cells (Figure 5H).

## 4 Discussion

Recent research increasingly views immunosenescence as a process of immune system adaptation or remodeling rather than solely harmful (Santoro et al. 2021). In our study, transcriptome analysis from childhood to old age revealed that while lymphocyte characteristics declined and inflammation and myeloid features increased rapidly in early life, these changes remained relatively stable during healthy elder aging. The stability of the transcriptome is correlated with the balance of immune cell numbers and functions, such as naïve T cells in healthy old individuals still exhibiting high expression of stemness and memory‐related genes (Galletti et al. 2020), which have been reported to be associated with maintaining healthy aging (Li et al. 2019). In addition, the transition from child to young adult was marked by significant transcriptional changes in peripheral immune cells, including a decline in B‐cell function and proportion, which could provide insights into age‐related infection susceptibility and vaccine application strategies (Siegrist and Aspinall 2009; Yang et al. 2021). Most previous studies investigated the transcriptional characteristics and composition of peripheral immune cells changes across different age groups (Ciocca et al. 2021; Harries et al. 2011; Luo et al. 2022; Terekhova et al. 2023); our study focused on covering a broader age range and exploring nonlinear changes in these features as was recently reported (Sparks et al. 2024). These findings emphasize the dynamic nature of the immune system during aging and provide important clues for understanding the mechanisms and patterns of immunosenescence.

The balance between pro‐ and anti‐inflammatory responses is crucial in healthy immunosenescence, as abnormal inflammation can disrupt immune homeostasis, leading to the intense inflammation observed in frail elderly individuals (Soysal et al. 2016). Our study observed a significant increase in the proportion of monocyte subpopulations (Mono_CD14‐S100A8) in frail individuals, which exhibit SASP characteristics and higher expression levels of inflammatory cytokine genes. In frail elderly individuals, PBMC subpopulations showed enrichment of inflammatory pathways, with Treg cells exhibiting significant TCR clonal expansion, activation, and effector function. IL‐6, a cytokine secreted by monocytes, was identified as a key regulator of Treg cells, particularly influencing eTreg differentiation (Raynor et al. 2015). The resulting immunosuppressive state by SASP‐phenotypic cells led to downregulation of cytotoxic cell function, impairing the clearance of abnormal senescent cells and creating a vicious cycle of inflammation (Eggert et al. 2016; Liu et al. 2023; Ruhland et al. 2016). Interestingly, we found that in healthy elderlies, cytotoxic cells showed higher expression of *FASLG* and *IFNG* compared with younger adults, consistent with previous reports (Bandrés et al. 2000; Zöphel et al. 2022). This may enhance the cytotoxic dynamics of NK and CD8^+^ T cells in the elderly (Le Garff‐Tavernier et al. 2010; Zöphel et al. 2022), enabling effective responses to infections, tumors, and other pathogens, contributing to healthy aging and potentially longer lifespans.

However, further investigation is needed to clarify the optimal balance between pro‐ and anti‐inflammatory activities across the lifespan, as well as to explore the role of neutrophils and solid organs in inflammatory aging. Nevertheless, our findings highlight the significant inflammation observed in frail individuals and suggest that targeting signaling pathways, particularly those involving interactions with pro‐ and anti‐inflammatory proteins like IL‐6 and Anxa1, could be promising strategies for anti‐aging and anti‐frailty interventions.

## 5 Conclusions

Our study revealed dynamic changes in the PBMC transcriptome across different ages, highlighting a shift from immune homeostasis in healthy elderlies to a dysregulated state in those with frailty. In frail individuals, we observed a marked disruption in the balance between pro‐ and anti‐inflammatory responses, along with a decline in cytotoxic function. Our study underscores the significance of monitoring immune variations throughout life, offering valuable insights for promoting healthy aging and preventing aging‐related diseases.

## Author Contributions

Wenpu Lai performed sequencing data analysis. Wenpu Lai, Wen Lei, Yangqiu Li, and Oscar Junhong Luo wrote the manuscript. Wen Lei, Chanchan Xiao, Yue Zhu, Zhenhua Li, Juan Wang, Yi Zhu, and Qiuyue Feng performed sample collection and experiments. Lipeng Mao and Jiacheng He assisted with the data interpretation. Hao Wang, Zhenhua Li, Guobing Chen, and Oscar Junhong Luo conceived the study, designed the experiments, and oversaw the research project.

## Conflicts of Interest

The authors declare no conflicts of interest.

## Supporting information


**Figure S1.** Age and batch distribution of PBMC RNA‐seq datasets. (A) Donor age distribution of PBMC RNA‐seq datasets. (B) Donor age distribution of PBMC RNA‐seq datasets for female (left) and male (right) group, respectively. (C) Comparison of donor ages between females and males. *p* Value calculated by T‐test (two‐sided). (D) PCA (Principal component analysis) visualization of PBMC RNA‐seq datasets before (left) and after (right) batch effect removal. Different batches are depicted with distinct colors. (E) Heatmap showing the correlation matrix of RNA‐seq samples before (left) and after (right) batch effect removal. (F) Comparison of the median expression levels of RNA‐seq samples across batches before (top) and after (bottom) batch effect removal. *p* Value calculated by Kruskal‐Wallis test. (G) Significantly enriched KEGG pathways for age‐associated genes. (H) Line graphs showing gene expression trajectories with age. Each line represents a gene. Gene expression levels were z scored, and trajectories were estimated by LOESS. (I) Significantly enriched KEGG pathways for each gene cluster in Figure 1D.
**Figure S2.** Characterization of PBMCs from healthy individuals by scRNA‐seq. (A) UMAP visualization of scRNA‐seq data from PBMCs of healthy individuals based on PCA (left) and SCALEX (right). Cells from different sample are depicted with distinct colors. (B) Same as A, but samples are colored based on datasets. (C) Same as A, but samples are colored based on major cell types. (D) Dot plot of marker genes for each cell type. The color and size of each dot represented the expression level and cell fraction of the marker genes, respectively. The number above the gray bar indicates the cell number of each cell type. The same applies to the rest of this figure. (E) Statistical comparison of cell cluster robustness in scRNA‐seq data using PCA vs. SCALEX for dimensionality reduction and clustering. Integration Local Inverse Simpson’s Index (iLISI) was employed as a metric to evaluate integration quality. Higher values indicate better batch effect removal performance. *p* value calculated by Wilcoxon test (two‐sided). (F) Left: UMAP visualization of myeloid cell clusters. Different cell types are depicted with distinct colors. Right: Dot plot of marker genes for each cell subtype. (G) Left: UMAP visualization of B‐cell clusters. Different cell types were depicted with distinct colors. Right: Dot plot of marker genes for each cell subtype. (H) Left: UMAP visualization of NK cell clusters. Different cell types were depicted with distinct colors. Right: Dot plot of marker genes for each cell subtype. (I) Left: UMAP visualization of T‐cell clusters. Different cell types were depicted with distinct colors. Right: Dot plot of marker genes for each cell subtype. (J) Boxplots for comparing the proportion of cell type for PBMCs between child and young adult sample groups. *p < 0.05, **p < 0.01, ***p < 0.001. Wilcoxon Rank Sum test (two‐sided).
**Figure S3.** TCRαβ clonotype characterization of T cells from healthy individuals. (A) Boxplots for comparing TCR repertoire clonal expansion (left) and Gini coefficient (right) of different T‐cell subsets of healthy individuals. (B) Heatmap visualization of pairwise transition likelihood (based on TCR clonotypes) between different T‐cell subsets. (C) Scatter‐plots of TCR clonal expansion (*y*‐axis) vs. age (*x*‐axis) for distinct T‐cell subtype. Fitted lines by polynomial function and confidence intervals (gray shaded area) are shown. (D) Same as C, but for Gini coefficient of TCR repertoire vs. age.
**Figure S4.** Comparison of PBMCs’ characteristics between healthy aging and frail elderlies. (A) Boxplots for comparing the age distribution between frail and healthy advanced‐aged individuals. *p* value calculated by t‐test (two‐sided). (B) Dotplots showing expression of selected genes in B_Naive‐IL4R cells from distinct age groups. (C) Significantly enriched GO biological process terms of upregulated genes in B_Naive‐IL4R cells of frail individuals in comparison with healthy advanced‐aged individuals. (D) Same as B, but for CD8_Naive cells. (E) Same as C, but for CD8_Naive cells. (F) The gating strategies of CD4^+^ and CD8^+^ T cells of PBMCs for aged and frail donors. (G) The gating strategies of CD45RA^+^CD62L^+^CD8^+^ T cells for aged and frail donors. (H) Bar plot showing percentage for CD45RA^+^CD62L^+^CD8^+^ T cells (naïve CD8^+^ T cells) of CD8^+^ T cells in aged and frailty. **p < 0.01. T‐test (two‐sided). (I) Violin plots showing SASP functional scores of monocytes across different age groups. ****p < 0.0001. Wilcoxon Rank Sum test (two‐sided).
**Figure S5.** Additional analysis of CD4^+^ T cells and cell–cell interactions. (A) Dotplot visualization of the ICOSL‐ICOS signal for each cell types of advanced‐aged and frail elderlies. The dot color and size represent the calculated communication probability and *p* values, respectively. (B) Dotplots showing expression of selected genes in conventional CD4^+^ T cells from distinct age groups. (C) Bar plot showing percentage for CD278^+^CD4^+^ T cells of CD4^+^ T cells in aged and frail donors. *p < 0.05. T‐test (two‐sided). (D) NicheNet analysis of the upregulated gene set in Treg cells (left: log_2_FC > 0.5, right: log_2_FC > 1) of frail compared with advanced‐aged donors. Heatmap shows the top 15 ligands with the highest predictive power for the gene expression Treg cells of frail donors. The Pearson’s correlation coefficient reflects the ability of each ligand to accurately predict the target genes in Treg cells. (E) Heatmap of NicheNet ligand–target matrix denoting the regulatory potential between the top 15 ligands of DEGs (log_2_FC > 0.75) and target genes from Treg cells from advanced‐aged donors. (F) Heatmap of NicheNet ligand–target matrix denoting the regulatory potential between the top 15 ligands and target genes from Treg cells from frail and advanced‐aged donors combined. (G) Bar plots showing the mean of outgoing information flow of IFN‐II signaling pathways for advanced‐aged and frail donors.

Table S1.
Table S2.
Table S3.
Table S4.
Table S5.
Table S6.
Table S7.
Table S8.
Table S9.
Table S10.

## Data Availability

Raw sequencing data of PBMCs RNA‐seq have been deposited in the Genome Sequence Archive in National Genomics Data Center, China National Center for Bioinformation/Beijing Institute of Genomics, Chinese Academy of Sciences (GSA‐Human: HRA006146) that are publicly accessible at https://ngdc.cncb.ac.cn/gsa‐human. The analyzed code for this study is available at https://github.com/aapupu/ImmunoRankAge.
