# Supplementary figures and images for: Deciphering Immunosenescence From Child to Frailty: Transcriptional Changes, Inflammation Dynamics, and Adaptive Immune Alterations
Source: Aging Cell. 2025 Apr 26;24(7):e70082. doi: 10.1111/acel.70082 (PMC12266785; doi:10.1111/acel.70082)

**Figure S1**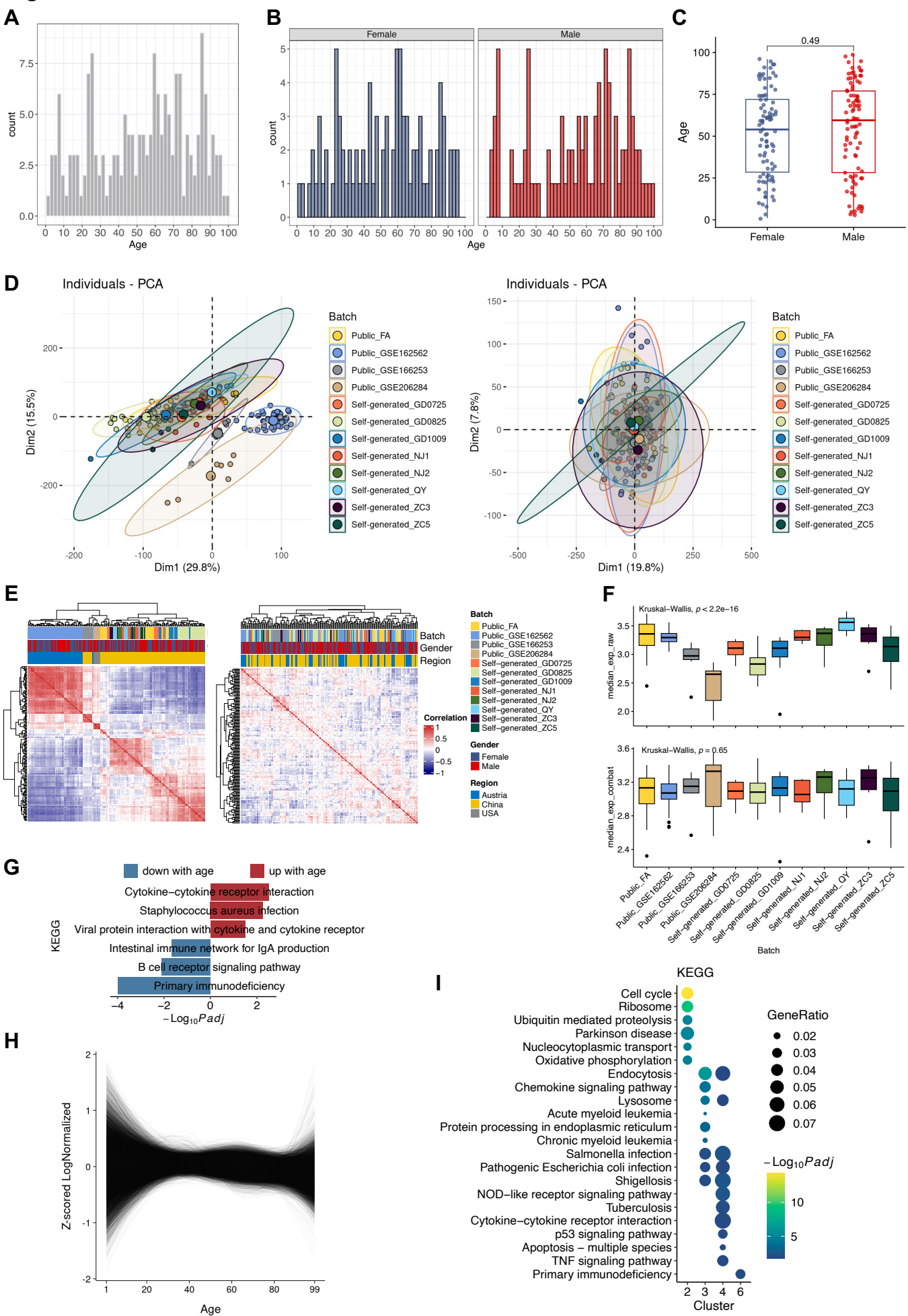

Figure S2

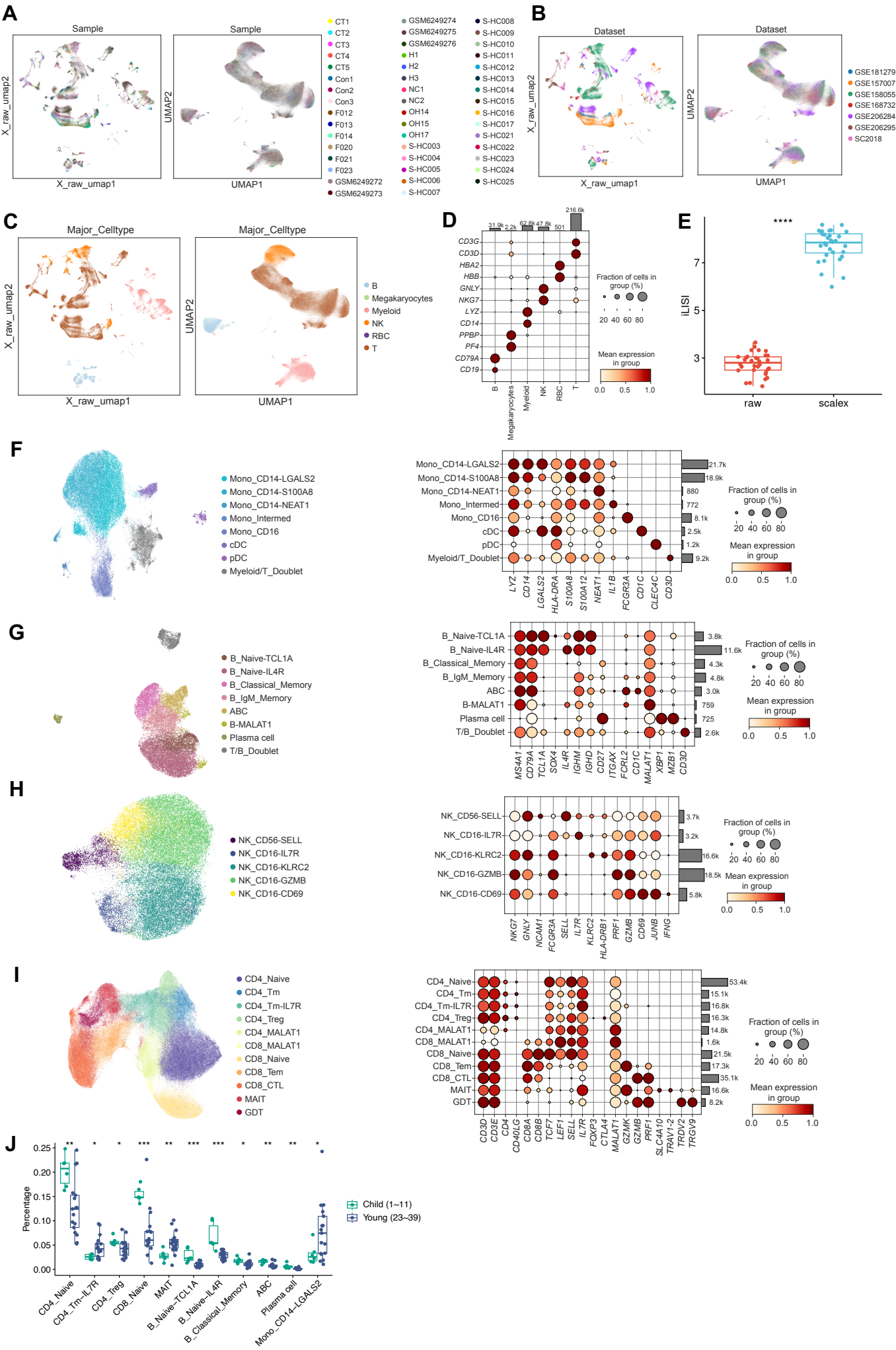

**Figure S3****A**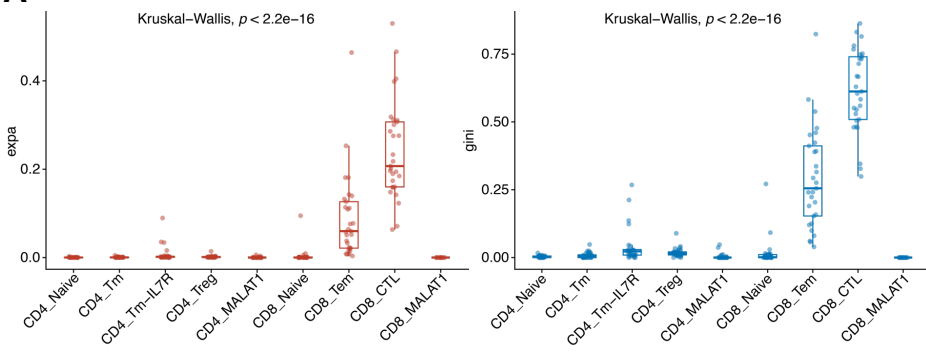**B**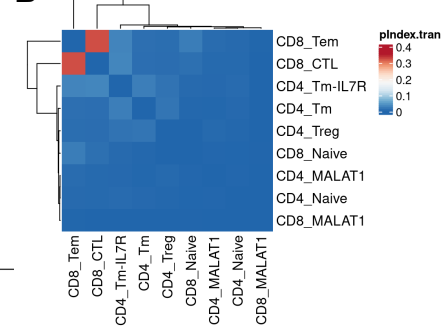**C**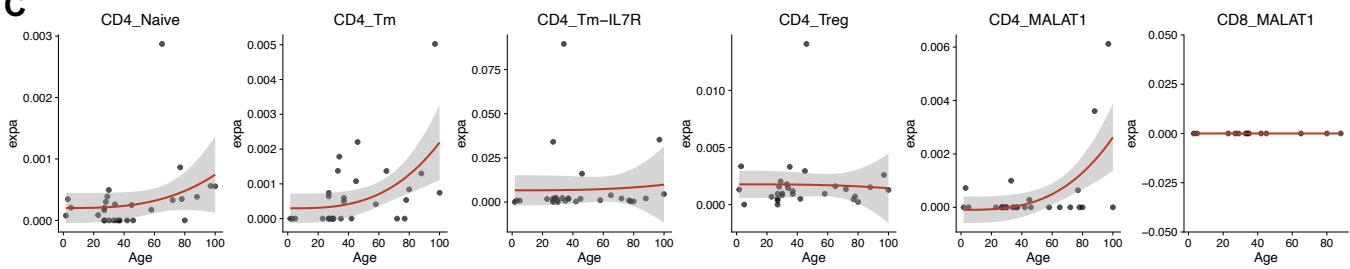**D**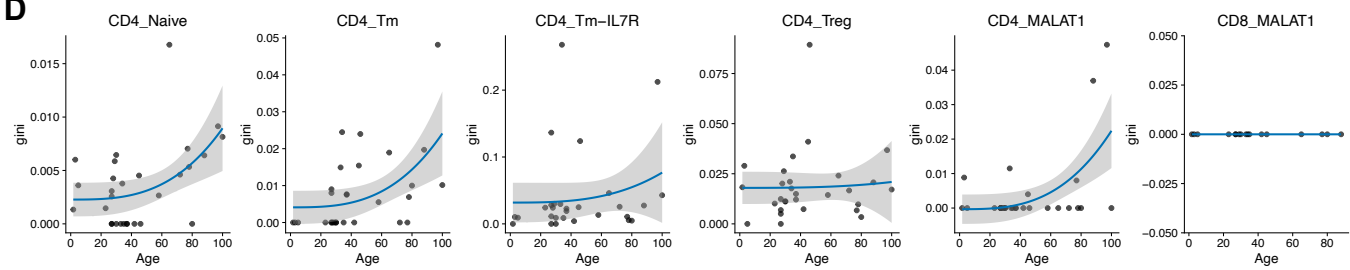

**Figure S4**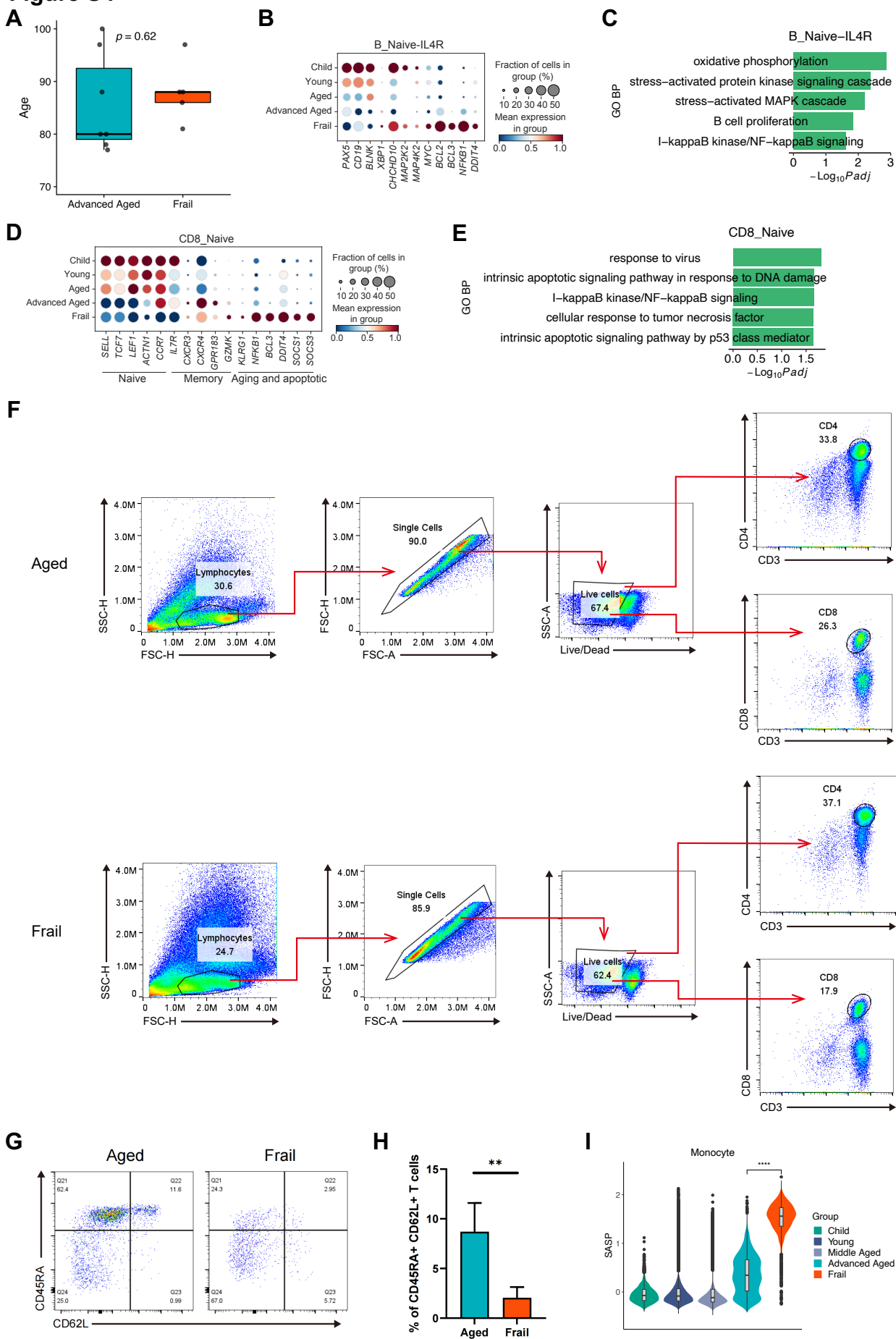

## A

Supplement: Supplementary file 1 — Figure S1. Age and batch distribution of PBMC RNA‐seq datasets. (A) Donor age distribution of PBMC RNA‐seq datasets. (B) Donor age distribution of PBMC RNA‐seq datasets for female (left) and male (right) group, respectively. (C) Comparison of donor ages between females and males. p Value calculated by T‐test (two‐sided). (D) PCA (Principal component analysis) visualization of PBMC RNA‐seq datasets before (left) and after (right) batch effect removal. Different batches are depicted with distinct colors. (E) Heatmap showing the correlation matrix of RNA‐seq samples before (left) and after (right) batch effect removal. (F) Comparison of the median expression levels of RNA‐seq samples across batches before (top) and after (bottom) batch effect removal. p Value calculated by Kruskal‐Wallis test. (G) Significantly enriched KEGG pathways for age‐associated genes. (H) Line graphs showing gene expression trajectories with age. Each line represents a gene. Gene expression levels were z scored, and trajectories were estimated by LOESS. (I) Significantly enriched KEGG pathways for each gene cluster in Figure 1D. Figure S2. Characterization of PBMCs from healthy individuals by scRNA‐seq. (A) UMAP visualization of scRNA‐seq data from PBMCs of healthy individuals based on PCA (left) and SCALEX (right). Cells from different sample are depicted with distinct colors. (B) Same as A, but samples are colored based on datasets. (C) Same as A, but samples are colored based on major cell types. (D) Dot plot of marker genes for each cell type. The color and size of each dot represented the expression level and cell fraction of the marker genes, respectively. The number above the gray bar indicates the cell number of each cell type. The same applies to the rest of this figure. (E) Statistical comparison of cell cluster robustness in scRNA‐seq data using PCA vs. SCALEX for dimensionality reduction and clustering. Integration Local Inverse Simpson’s Index (iLISI) was employed as a metric to ev [file ACEL-24-e70082-s001.pdf]
